# Varying Inundation Regimes Differentially Affect Natural and Sand-Amended Marsh Sediments

**DOI:** 10.1371/journal.pone.0164956

**Published:** 2016-10-27

**Authors:** C. Wigand, K. Sundberg, A. Hanson, E. Davey, R. Johnson, E. Watson, J. Morris

**Affiliations:** 1 Atlantic Ecology Division, ORD-NHEERL, U.S. Environmental Protection Agency, Narragansett, Rhode Island, United States of America; 2 Belle W. Baruch Institute for Marine and Coastal Sciences and Department of Biological Sciences, University of South Carolina, Columbia, South Carolina, United States of America; Centro de Investigacion Cientifica y de Educacion Superior de Ensenada Division de Fisica Aplicada, MEXICO

## Abstract

Climate change is altering sea level rise rates and precipitation patterns worldwide. Coastal wetlands are vulnerable to these changes. System responses to stressors are important for resource managers and environmental stewards to understand in order to best manage them. Thin layer sand or sediment application to drowning and eroding marshes is one approach to build elevation and resilience. The above- and below-ground structure, soil carbon dioxide emissions, and pore water constituents in vegetated natural marsh sediments and sand-amended sediments were examined at varying inundation regimes between mean sea level and mean high water (0.82 m NAVD88 to 1.49 m NAVD88) in a field experiment at Laws Point, part of the Plum Island Sound Estuary (MA). Significantly lower salinities, pH, sulfides, phosphates, and ammonium were measured in the sand-amended sediments than in the natural sediments. In natural sediments there was a pattern of increasing salinity with increasing elevation while in the sand-amended sediments the trend was reversed, showing decreasing salinity with increasing elevation. Sulfide concentrations generally increased from low to high inundation with highest concentrations at the highest inundation (i.e., at the lowest elevations). High pore water phosphate concentrations were measured at low elevations in the natural sediments, but the sand-amended treatments had mostly low concentrations of phosphate and no consistent pattern with elevation. At the end of the experiment the lowest elevations generally had the highest measures of pore water ammonium. Soil carbon dioxide emissions were greatest in the sand-amended mesocosms and at higher elevations. Differences in coarse root and rhizome abundances and volumes among the sediment treatments were detected with CT imaging, but by 20 weeks the natural and sand-amended treatments showed similar total belowground biomass at the intermediate and high elevations. Although differences in pore water nutrient concentrations, pH, salinity, and belowground root and rhizome morphology were detected between the natural and sand-amended sediments, similar belowground productivity and total biomass were measured by the end of the growing season. Since the belowground productivity supports organic matter accumulation and peat buildup in marshes, our results suggest that thin layer sand or sediment application is a viable climate adaptation action to build elevation and coastal resiliency, especially in areas with low natural sediment supplies.

## Introduction

Accelerated sea level rise and an increase in the frequency and severity of storms are predicted for some regions of the US due to climate change [1,2,3,4]; these stressors can have negative impacts on tidal marsh systems because they can increase flooding and erosion [5,6,7,8,9]. Accelerated sea level rise and/or low sediment inputs have been shown to contribute to coastal marsh loss [10,11,12,13]. These factors can result in large expanses of coastal wetlands that may require restoration and climate-adaptation actions, including but not limited to: shoreline protection, wetland reconstruction, and in some cases, sediment additions to the surface of the marsh platform to restore and build coastal resiliency [14,15,16,17].

Large-scale sediment amendments to disappearing marshes in Jamaica Bay, NY have been carried out and the marshes appear to be on a successful trajectory for restoring marsh ecosystem services [18,19,20,21,22]. Similar efforts to restore marsh elevation with thin-layer deposition of dredged materials have been carried out in the Mississippi River delta [23,24] and more recently in the mid-Atlantic (i.e., Delaware [25]) and northeast (i.e., Rhode Island [26]). Managers and restoration specialists need to consider the appropriate layer thickness for sediment application, the type of sediment to apply, and the location for placement of materials on deteriorating coastal wetlands [15]. Researchers [27] examined the effects of varying dredged sediment additions, ranging from trace amounts to greater than 30 cm of slurry additions, on plant community structure and sediment characteristics in a rapidly subsiding and deteriorating marsh in the Gulf of Mexico. Plant biomass increased with increasing sediment slurry deposition, and the sediment additions heightened marsh elevation. The increase in elevation as a result of the sediment subsidies reduced the depth of flooding, improved soil aeration, and reduced the concentration of phytotoxins, such as sulfides [14,27].

**Fig 1:**
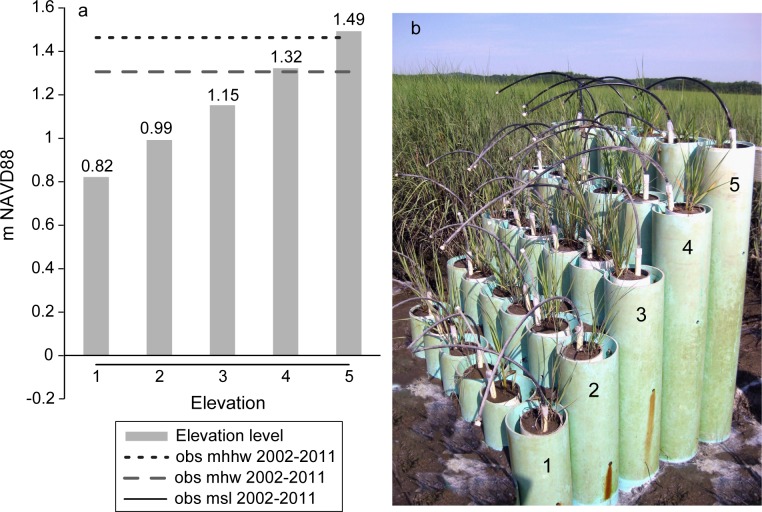
(a) Elevation of natural and sand-amended mesocosms ranging from 0.82 m NAVD88 to 1.49 m NAVD88 and treatment levels from lowest (elevation 1) to highest (elevation 5). Mean high water (mhw), mean high high water (mhhw) and mean sea level (msl) observed at nearby Portland ME observed for the period 2002–2011 are indicated for reference. (b) Natural and sand-amended mesocosms with inserts and lysimeters, and elevation levels labelled.

In our study we compared the effect of varying inundation regimes on sand-amended and natural marsh sediments in a field experiment. Marsh plant productivity responds to variations in the duration and frequency of flooding, and the response is dependent upon the optimal elevation for growth within the tidal frame [28]. Marsh mesocosms were set at different elevations between mean sea level and mean high water in the field, and therefore subjected to different inundation treatments (Fig 1). We measured above- and below-ground plant biomass, pore water chemistry, and carbon dioxide efflux of both natural and sand-amended marsh sediments. Inundation increases were hypothesized to alter sediment biogeochemistry differentially in natural and sand-amended sediments, and changes in the soil biogeochemistry were hypothesized to have an adverse effect on plant growth under some sediment and inundation conditions. Previous studies have demonstrated negative effects on above- and below-ground production under some elevated inundation regimes [29,30,31], with greatest adverse effects of high inundation in sediments with low organic matter [32]. However, marsh plants have been shown to have nonlinear growth patterns with optimal growth at intermediate marsh elevations within the tidal frame, and elevations below and above this level having reduced production [28,33]. Whereas vegetation above the optimal elevation is expected to increase in productivity with sea level rise, plants below the optimal elevation are expected to decline with increased inundation [28]. In marsh soils, under waterlogged and high nutrients conditions, sulfides can accumulate and concentrations >1 mM can reduce *S*. *alterniflora* growth by interfering with metabolic processes including ammonium uptake [34,35]. Resource managers faced with drowning coastal marshes and opting for sand or sediment application to build marsh resiliency will benefit from understanding responses to sand amendments at different inundation levels, as undertaken in this study.

## Methods

### Experimental design

The study was conducted from 27 April to 15 September 2011 (141 days) at Laws Point, part of the Plum Island Long Term Ecological Research Site (MA) [28]. The tidal range at Plum Island averages about 2.67 m. The marsh planter, referred to as a “marsh organ’, consisted of 30 PVC pipes (15-cm diameter) of five different heights set in rows of six pipes per row at the marsh edge [28]. The elevation of the top surface of the pipes differed by 16–17 cm between rows; the 5 experimental elevations were 0.82, 0.99, 1.15, 1.32 and 1.49 m relative to the North American Vertical Datum of 1988 (NAVD 88) (Fig 1A and 1B). Henceforth, we refer to the elevations as levels 1, 2, 3, 4, and 5 ranging from lowest 0.82 m NAVD 88 to highest 1.49 m NAVD 88, respectively. The elevation range spanned from 47 cm below to 17 cm above mean high water (mhw). The design of the marsh organ allows us to approximate how plants are expected to respond to increasing periods of inundation and vertical exchanges of water [29,36]. We recognize, however, that lateral exchanges of water and soil drainage were different in the marsh organ than found in natural marsh systems. However, even with these limitations the marsh organ allows for approximating conditions influencing marshes as they respond to sea level rise [36].

The PVC pipes were filled with natural marsh sediment collected from a nearby creek, except for the top 40 cm. PVC inserts (40 cm long and 10 cm in diameter) were fitted with a 1-mm mesh bottom and placed into the top of the pipes such that the mesh rested on the sediment that had previously been placed in the pipes. Half of the 30 inserts were filled with the natural marsh sediments, and half were filled with a sand/natural sediment mix (vol:vol, 3:1). The sand was untreated and collected from a nearby quarry (Middleboro, MA) and mixed with the natural sediments with a hand shovel at the marsh site. Bulk density, percent organic matter (OM), and porosity were determined on 5 subsamples each of the natural sediment and the sand/natural sediment mix to characterize the soil. The bulk density of the sand amended sediments was 1.79 ± 0.18 g ml^-1^ and the OM, 1.65 ± 0.14%. The bulk density of the sand amended sediments was about 2.8 X higher than the natural marsh sediments (0.65 ± 0.06 g ml^-1^) and 5X less organic than the natural sediments (8.8 ± 0.43% OM). The sand-amended soil porosity was estimated at about 31% and the natural soil porosity at 74% using the formula described by Callaway et al. [37].

One field-collected *Spartina alterniflora* plug (5 cm X 15 cm) was planted into each PVC insert on 26 April 2011. At each of the five elevations, three each of the natural sediment and sand-amended inserts were placed into the pipes, and the order of pipes in each row was assigned randomly. Each individual pipe plus insert represented an experimental unit or mesocosm.

### Above- and below-ground morphology and biomass

Shoot height and density were measured monthly throughout the growing season, from May to September. Measurements were taken in late May, and no height and density data were recorded in June. Standing aboveground biomass was harvested at the end of the growing season in each mesocosm. Plant stems were clipped, rinsed, and dried to a constant weight at 60°C. Roots and rhizomes were separated from one half of the sediment by rinsing and hand-sieving with a 500 μm sieve, and then dried at 60°C to estimate belowground biomass.

### Computer-aided Tomography

The PVC inserts were imaged using computer-aided tomography (CT) in July at the middle (10 weeks) and in September at the end (20 weeks) of the field experiment. The inserts were removed, scanned, and, in July, returned to the organ within 24 hours. Inserts were scanned in a GE Medical Systems model Light Speed 16 CT Scanner (Milwaukee, Wisconsin, USA) at an energy setting of 120 kilovolts and 215 milliamps. We were able to quantify the volume, abundances, and diameters of coarse roots and rhizomes using the estimated densities for each soil component as defined by calibration rods of water, air, and glass [38,39], and with ImageJ, a public domain, Java-based image processing program developed at the National Institutes of Health [40]. The coarse roots were defined as having diameters of greater than or equal to 1 mm but less than 2 mm, and rhizomes were defined as having diameters greater than or equal to 2 mm [41]. The CT image analyses had enough resolution to quantify roots greater than 1 mm in width using this method. Abundances of coarse roots and rhizomes (# m^-2^) were reported for 0-10 cm sediment depth, a sediment depth with active *S*. *alterniflora* root and rhizome growth [38]. Volumetric measures (mm^3^) of belowground root and rhizomes to a depth of 30 cm were also determined for a more comprehensive estimate of belowground biomass. Following the quantitative analyses, the CT data for each was downloaded and photographic images were created using Osirix Imaging Software (http://www.osirix-viewer.com/). In Osirix, a Sharpen 5x5 convolution kernel filter and auto-segmentation were used for the creation of the root images.

### Soil carbon dioxide emission rates

Soil carbon dioxide emissions from the mesocosms with clipped vegetation were measured at the end of the experiment with a Li-Cor (8100) CO_2_ flux system and dome using standard methods [42]. The Li-Cor instrument uses an infrared detector to measure changes in carbon dioxide in the dome within short (5 minute) incubations. Cut stems were plugged with silicone gel to prevent gas exchange through lacunae [42].

### Pore water chemistry

Ambient pore water was collected monthly (July–August) from each mesocosm at a depth 20 cm depth using a permanently-installed micropiezometer outfitted with a 0.5 μm nylon mesh frit. Unfiltered samples were preserved in the field for hydrogen sulfide analysis with 0.22% zinc acetate (Zn(O_2_CCH_3_)_2_) solution (vol:vol, 1:1). Pore water was analyzed for pH and salinity, then preserved for orthophosphate and ammonia analyses, by filtering and preserving with sulfuric acid (120μL 6M H_2_SO_4_:20 ml pore water). These samples were later analyzed for hydrogen sulfide, orthophosphate and ammonium using spectrophotometric methods [43,44]. Hydrogen sulfide levels < 0.5 μM were not detectable using these methods and recorded as 0.

### Statistics

Two-way ANOVA models were used to examine for the main effects of sediment type and elevation and sediment X elevation interaction on above- and below-ground structure, soil carbon dioxide emission rates, and pore water analytes. Nutrient pore water concentrations were natural log-transformed to meet ANOVA assumptions. When the ANOVA models were significant, differences among treatment means were tested with a protected Fisher’s least significant difference (LSD) post-hoc test. The probability for significance was P < 0.05 for all statistical analyses.

## Results

### Aboveground responses

**Fig 2:**
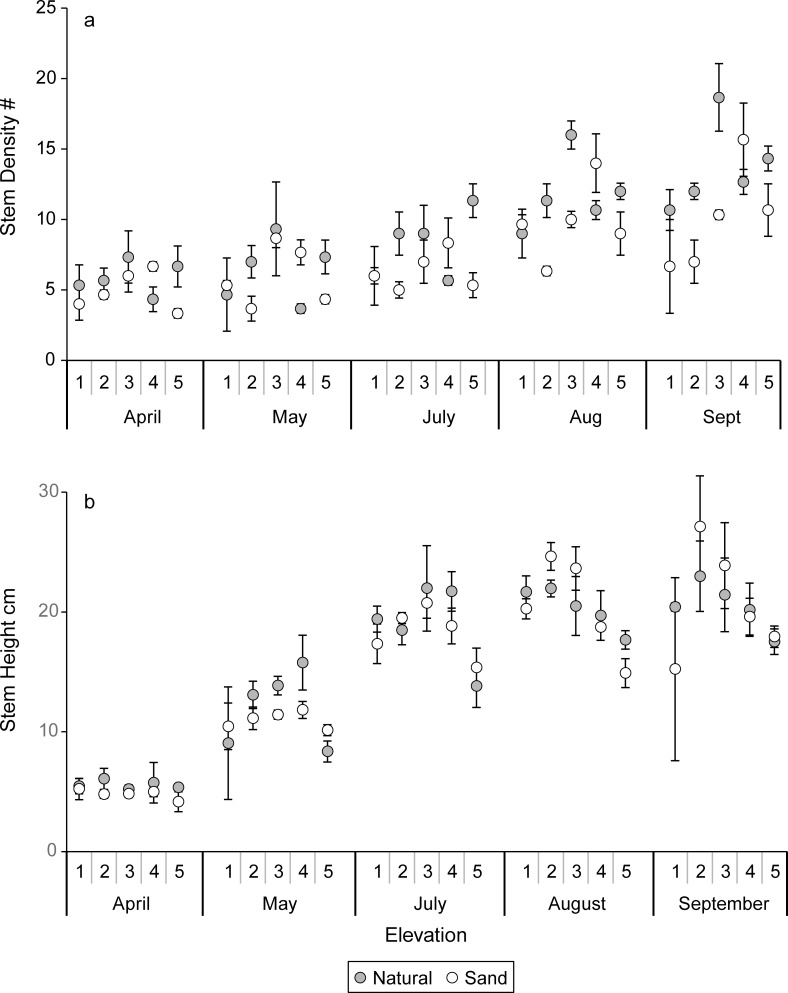
(a) Mean stem densities (# per mesocosm) and standard errors and (b) mean stem heights (cm) and standard errors in natural and sand-amended mesocosms for elevation levels 1–5 for April–September.

The stem density and stem height in April, May, and July were similar between the sand-amended and natural marsh sediments with no significant main effects of sediment or elevation nor significant sediment X elevation interactions, indicating samples were initially homogenous (Fig 2A and 2B). In August there was a significant (P = 0.007) main effect of elevation and a significant (P = 0.004) sediment X elevation interaction on stem density, while the sand-amended sediments also had significantly lower stem density (Table 1). The natural marsh sediments in August (P = 0.01) and September (P = 0.006) (Fig 2A, Table 1). Stem densities were greatest at an intermediate elevation (3), significantly greater than at elevations 5, 1, and 2 in August. Intermediate elevations (3, 4) again had significantly (P = 0.01) greater stem densities in September, but no significant sediment X elevation interaction (Table 1).

**Table 1:** ANOVA model results for stem density and height with significant (P < 0.05) main effects of elevation. Means ± standard error (SE) are shown for each. Significant sediment X elevation interactions are indicated with an asterisk. Main effects of sediment are indicated. Means with different letters are significantly different (P < 0.05), ns is non-significant.

	*Stem Density (#) August				Stem Density (#) Sept				Stem Height (cm) August			
Soil Matrix	Mean		SE		Mean	SE			Mean		SE	
Natural	11.80	±	0.75	A	13.67	±	0.91	A	20.30	±	0.74	ns
Sand	9.80	±	0.81	B	10.07	±	1.20	B	20.44	±	1.04	
**Elevation**												
1	9.33	±	0.84	C	8.67	±	1.86	C	20.97	±	0.77	AB
2	8.83	±	1.25	C	9.50	±	1.34	BC	23.31	±	0.85	A
3	13.00	±	1.44	A	14.50	±	2.16	A	22.08	±	1.54	AB
4	12.33	±	1.23	AB	14.17	±	1.40	A	19.23	±	0.96	BC
5	10.50	±	0.99	BC	12.50	±	1.23	AB	16.29	±	0.89	C

**Fig 3:**
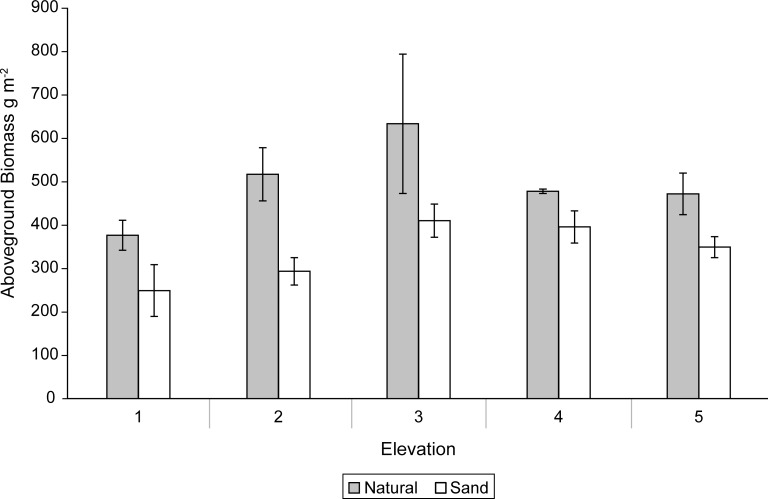
Harvested mean aboveground biomass (g m^-2^) and standard errors for natural and sand-amended mesocosms at elevation levels 1–5 in September, at the end of experiment.

We did not detect a main effect of sediment nor significant sediment X elevation interaction on stem height in August and September (Fig 2B). However, a significant (P = 0.0008) main effect of elevation on stem height was detected in August but not September (Fig 2B, Table 1). Stem heights were greatest at intermediate elevations (2, 3) in August (Fig 2B, Table 1). The natural marsh sediments had 31.5% greater (P = 0.001) aboveground biomass than the sand-amended sediments in September (Fig 3). An elevation trend (P = 0.06) was detected with greatest aboveground biomass at intermediate elevations, but no significant sediment X elevation interaction.

### Belowground responses

#### CT coarse root and rhizome abundances, volumes, and diameters

**Fig 4:**
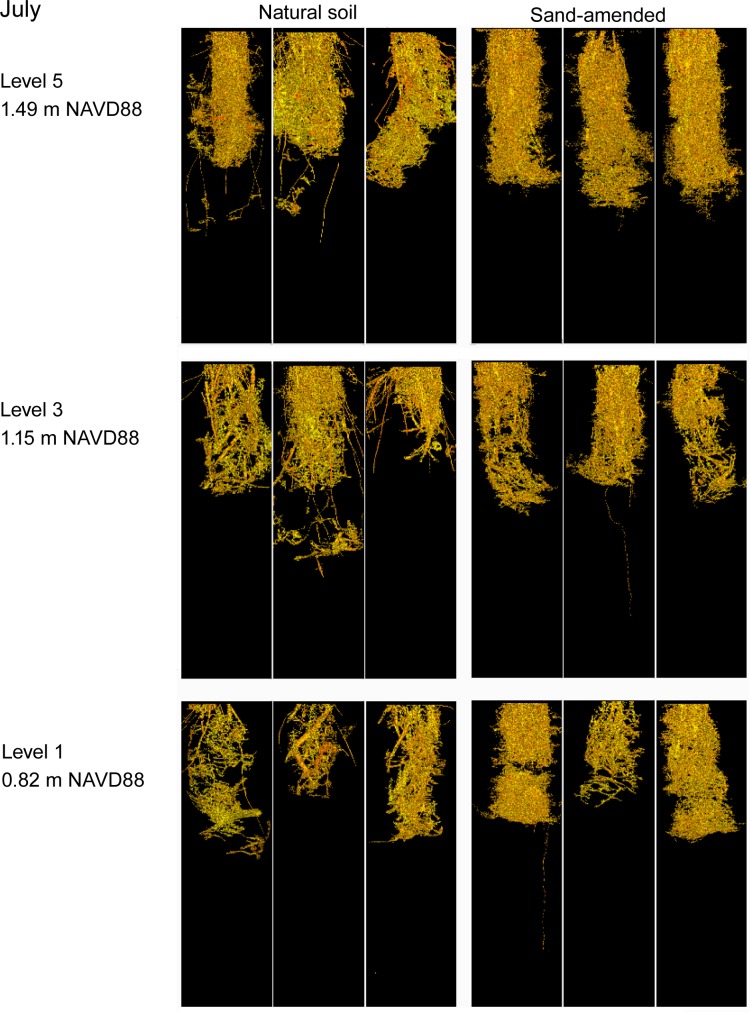
Computer-aided tomography images of natural and sand-amended mesocosms (0–30 cm depth) at elevation levels 1, 3, and 5 in July, 10 weeks after planting.

**Fig 5:**
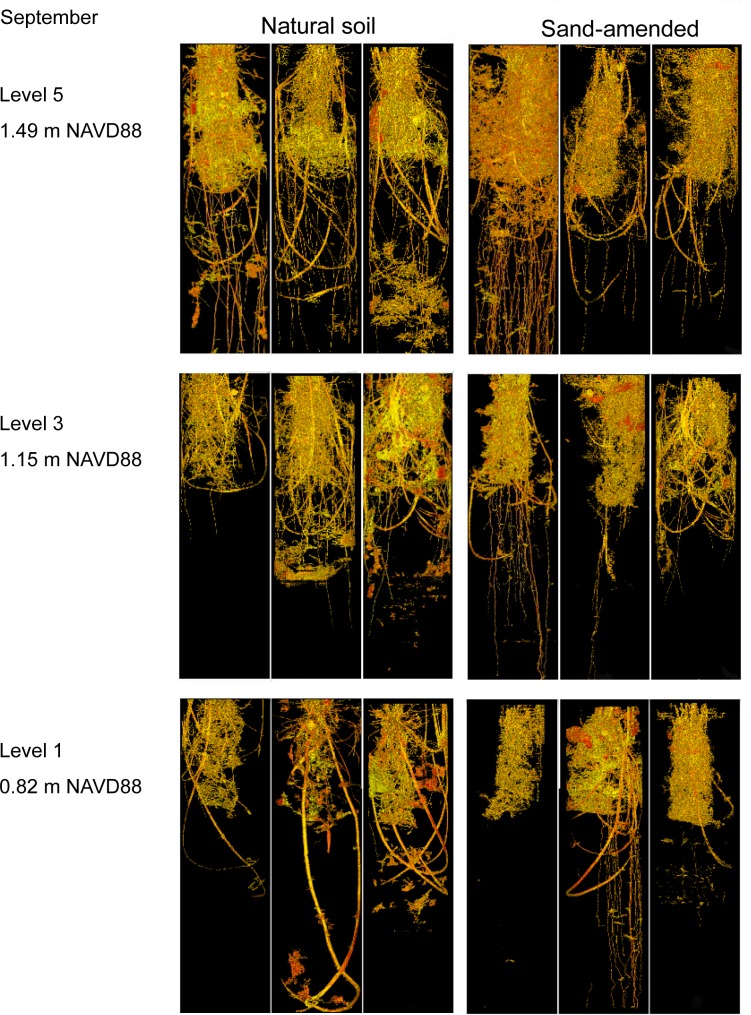
Computer-aided tomography images of natural and sand-amended mesocosms (0–30 cm depth) at elevation levels 1, 3, and 5 in September, 20 weeks after planting.

**Fig 6:**
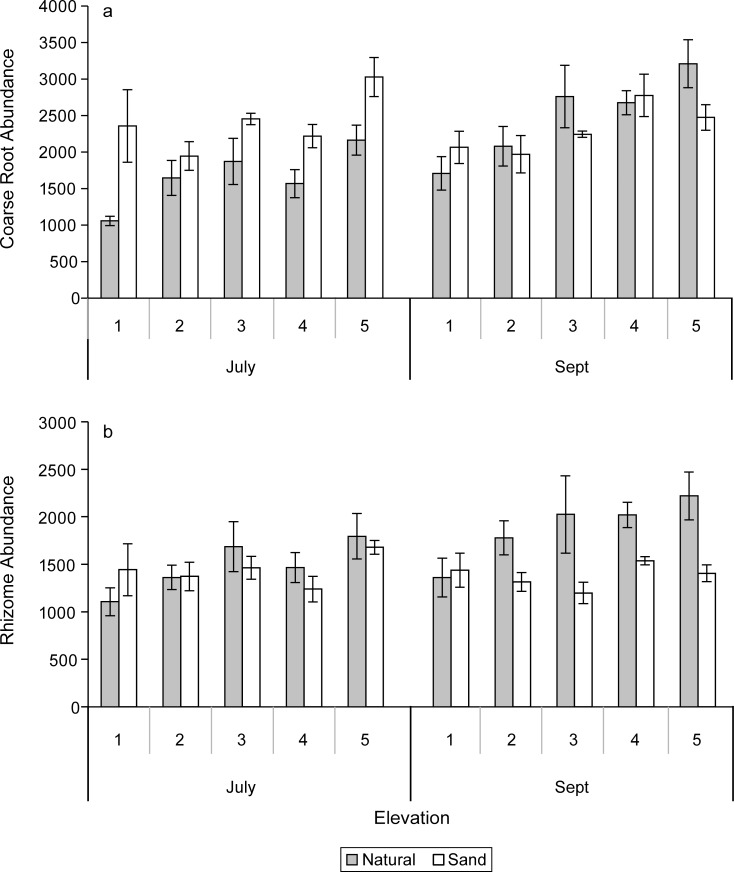
Mean abundances (# m^-2^) and standard errors of (a) coarse roots and (b) rhizomes in the upper 10 cm of the mesocosms determined with computer-aided tomography in July, 10 weeks after planting and (b) in September, 20 weeks after planting for elevation levels 1–5.

No significant sediment X elevation interactions were measured for coarse root abundances in either July or September. The sand-amended treatment showed significantly (P = 0.0001) higher abundances of coarse roots than the natural sediments in July, but the abundances of coarse roots were similar among the sand-amended and natural sediments by September (Figs 4, 5 and 6A). The coarse root abundances were greatest at the highest elevation (5), which was similar to abundances at elevations 4 and 3, but significantly (P = 0.004) greater than abundances at lower elevations (2, 1) in September (Table 2). The rhizome abundances were similar between the natural and sand-amended treatments in July, but the rhizome abundances were significantly (P = 0.0006) greater in the natural than the sand-amended treatments in September (Fig 6B). The main effect of elevation and the sediment X elevation interaction on rhizome abundances were not significant in September.

**Table 2:** ANOVA model results for computer-aided tomography (CT) estimates of coarse root abundances (0–10 cm), coarse root volumes (0–30 cm), and coarse root diameters with significant (P < 0.05) main effects of elevation. Means ±standard error (SE) are shown for each. Significant sediment X elevation interactions are indicated with an asterisk. Main effects of sediment are indicated. Means with different letters are significantly different (P < 0.05), ns is non-significant.

	July CT Root Abundance (# m^-2^)				Sept CT Root Abundance (# m^-2^)				*July CT Root Vol (mm^3^)				Sept CT Root Vol (mm^3^)				*July CT Root Diam (mm)			
Soil Matrix	Mean		SE		Mean		SE	Mean		SE		Mean		SE		Mean		SE	
Natural	1661.3	±	123.8	B	2487.1	±	174.7	ns	5499.6	±	230.4	ns	6177.7	±	357.2	A	1.457	±	0.005	A
Sand	2401.2	±	137.3	A	2306.2	±	108.6		5316.2	±	325.7		5370	±	353.6	B	1.423	±	0.005	B
**Elevation**																				
1	1707.8	±	334.9	B	1886.6	±	148.7	C	4513.3	±	295.0	B	4423.5	±	264.6	D	1.458	±	0.010	A
2	1795.4	±	140.5	B	2025.3	±	153.1	BC	5209.8	±	252.6	B	4898.4	±	310.0	CD	1.443	±	0.004	AB
3	2162.6	±	178.4	AB	2502.3	±	204.3	AB	5394.4	±	378.0	B	5540.2	±	538.5	BC	1.448	±	0.013	A
4	1894.3	±	167.8	B	2726.4	±	137.6	A	5180.9	±	287.2	B	6467.5	±	307.8	AB	1.428	±	0.009	BC
5	2596.2	±	223.4	A	2842.6	±	213.2	A	6741.1	±	425.1	A	7539.7	±	350.8	A	1.425	±	0.008	C

**Fig 7:**
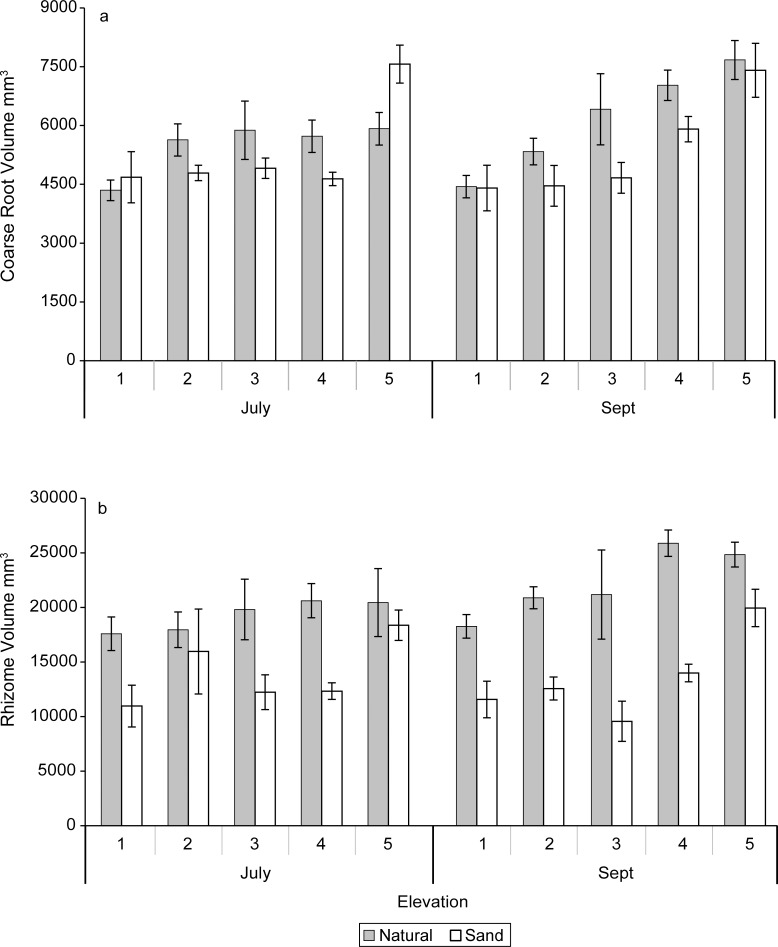
Mean volumes (mm^3^) and standard errors of (a) coarse roots and (b) rhizomes in the upper 30 cm of the mesocosms determined with computer-aided tomography in July, 10 weeks after planting and (b) in September, 20 weeks after planting for elevation levels 1–5.

In July, there was a significant interaction between sediment X elevation (P = 0.02) for coarse root volume (0–30 cm), and the main effects measured no significant difference between the natural sediments and sand-amended, but the highest elevation coarse root volumes were significantly greater than those in all other elevation treatments (P = 0.001). Sand-amended treatments had greater volumes of coarse roots than the natural sediments at the highest elevation (5), while natural sediments exceeded or were equal to sand-amended treatment volumes at all other elevations in July (Fig 7A).

The coarse root volumes of the natural sediments were significantly (P = 0.02) greater than the sand-amended ones in September (Fig 7A, Table 2). Elevation 5 had a significantly (P < 0.0001) greater volume of coarse roots than elevations 3, 2, and 1, and there was no significant sediment X elevation interaction in September (Table 2). The rhizome volumes were significantly greater in the natural marsh sediments than sand-amended in July (P = 0.001) and September (P < 0.0001) (Fig 7B), but there was no significant main effect of elevation nor a sediment X elevation interaction.

The coarse root diameters had a significant elevation X sediment interaction (P = 0.04) in July, and the diameters were significantly greater in the natural marsh sediments than the sand-amended sediments in July (P < 0.0001, Table 2), but similar in magnitude in September. Generally, there was a pattern of greater coarse root diameters at lower elevations (except elevation 2) than at higher elevations in July (P = 0.003; Table 2). In contrast, neither sediment nor elevation treatments had an effect on the rhizome diameters in either July (mean diameter: 3.178 ± 0.024 mm, n = 30) or September (mean diameter: 3.058 ± 0.023 mm, n = 30).

#### Belowground biomass determined by hand-sieving

**Fig 8:**
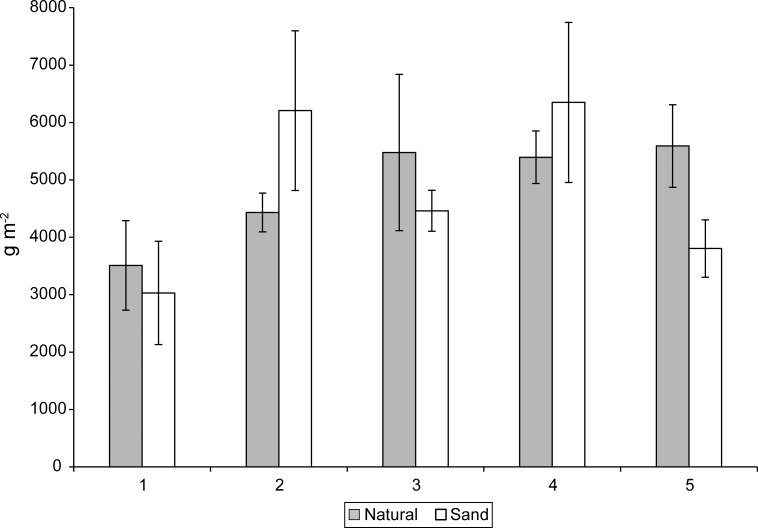
Hand-sieved mean belowground biomass (g m^-2^) and standard errors for natural and sand-amended mesocosms at elevation levels 1–5 in September, at the end of experiment.

We found no significant main effects of sediment or elevation nor a significant sediment X elevation interaction on the total dry belowground biomass as determined by hand-sieving (Fig 8). Total belowground biomass included fine roots (< 1mm in diameter), coarse roots, and rhizomes. Since we did detect significantly greater volumes (0–30 cm depth) of coarse roots and rhizomes and significantly greater abundances of rhizomes after 20 weeks (September) in the natural sediments by CT imaging, we suspect the sand-amended treatments had greater volumes of fine roots. We could not quantitatively measure fine roots (< 1mm) with CT imaging because of resolution limitations, but our observations during hand-sieving and in the CT qualitative images (Fig 5) suggest greater fine roots in the sand-amended treatments than the natural sediments after 20 weeks.

#### CO_2_ efflux

**Fig 9:**
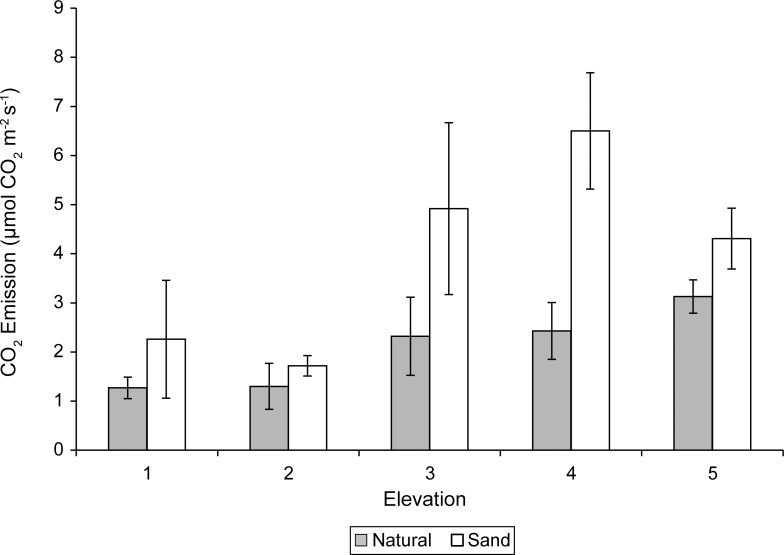
Mean soil carbon dioxide emissions (μmol CO_2_ m^-2^ s^-1^) and standard errors of natural and sand-amended mesocosms at elevation levels 1–5 in September, at the end of the experiment.

We detected significant main effects of sediment (P = 0.003) and elevation (P = 0.01), but no significant sediment X elevation interaction on soil carbon dioxide emission rates. CO_2_ efflux rates were 47% greater in the sand-amended treatments than the natural marsh sediments and the greatest emission rates were at the higher elevations (≥ elevation 3) (Fig 9).

#### Pore water

**Fig 10:**
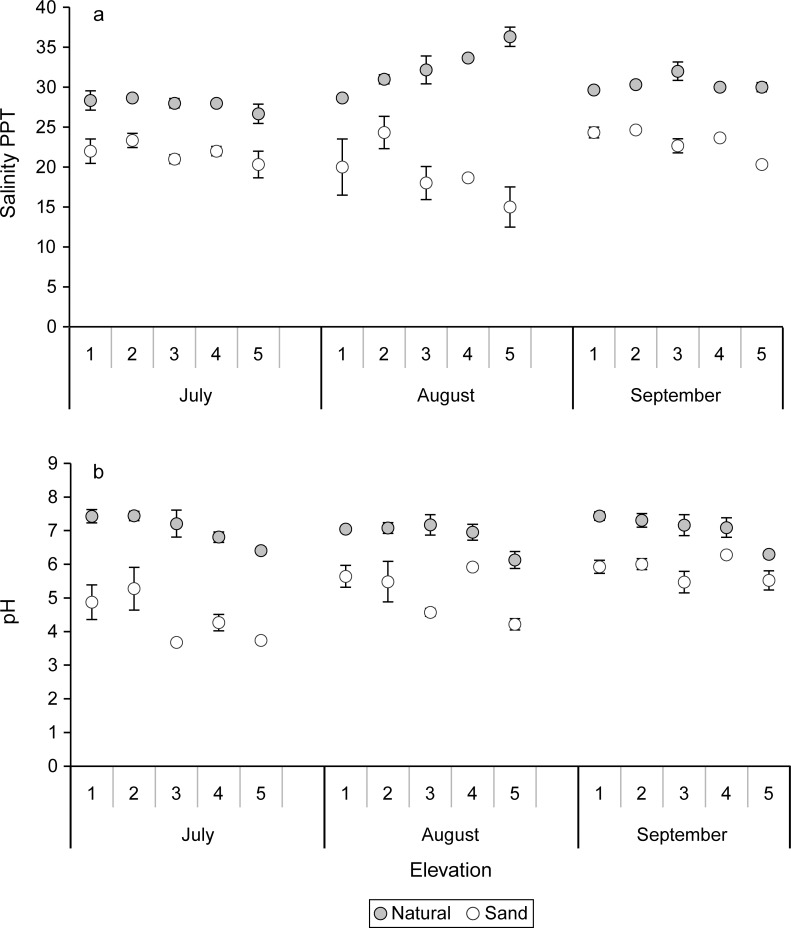
Means and standard errors of (a) pore water salinity (ppt) and (b) pH for natural and sand-amended mesocosms at elevation levels 1–5 for July, August, and September.

The salinity and pH in the pore water of the sand-amended treatments were significantly (P < 0.0001) lower than those concentrations measured in the pore water of the natural marsh sediments in July, August, and September (Fig 10A and 10B). There were significant (P ≤ 0.005) sediment X elevation interactions on salinity in August and September. In the natural sediments there was a pattern of increasing salinity with increasing elevation while in the sand-amended sediments the trend was reversed, decreasing salinity with increasing elevation, especially noticeable in August. However, in July (natural 27.9 ± 0.4 ppt; sand 21.7 ± 0.5 ppt, n = 15 each) and August (natural 32.4 ± 0.8 ppt; sand 19.2 ± 1.2 ppt, n = 15 each) there were no main effects of elevation on the salinity, but in September the highest elevation (5) had the lowest salinity, mostly driven by the sand-amended sediments (Fig 10A, Table 3). In all three months there were significant (P < 0.01) main effects of elevation on pH and the highest elevation (5) had the lowest pH (Fig 10B).

**Table 3:** ANOVA model results for salinity and pH with significant (P < 0.05) main effects of elevation. Means ± standard error (SE) are shown for each. Significant sediment X elevation interactions are indicated with an asterisk. Main effects of sediment are indicated. Means with different letters are significantly different (P < 0.05), ns is non-significant.

	Sept Salinity* (ppt)				July pH				August pH				Sept pH			
Soil Matrix	Mean		SE		Mean		SE		Mean	SE			Mean		SE	
Natural	30.40	±	0.32	A	7.06	±	0.14	A	6.88	±	0.13	A	7.06	±	0.14	A
Sand	23.13	±	0.47	B	4.37	±	0.22	B	5.17	±	0.21	B	5.84	±	0.12	B
**Elevation**																
1	27.00	±	1.24	A	6.15	±	0.62	AB	6.35	±	0.35	A	6.68	±	0.35	A
2	27.50	±	1.28	A	6.36	±	0.56	A	6.28	±	0.45	A	6.66	±	0.31	A
3	27.33	±	2.19	A	5.45	±	0.81	C	5.87	±	0.60	A	6.32	±	0.43	AB
4	26.83	±	1.42	A	5.54	±	0.58	BC	6.44	±	0.26	A	6.68	±	0.22	A
5	25.17	±	2.18	B	5.07	±	0.60	C	5.17	±	0.45	B	5.91	±	0.22	B

**Fig 11:**
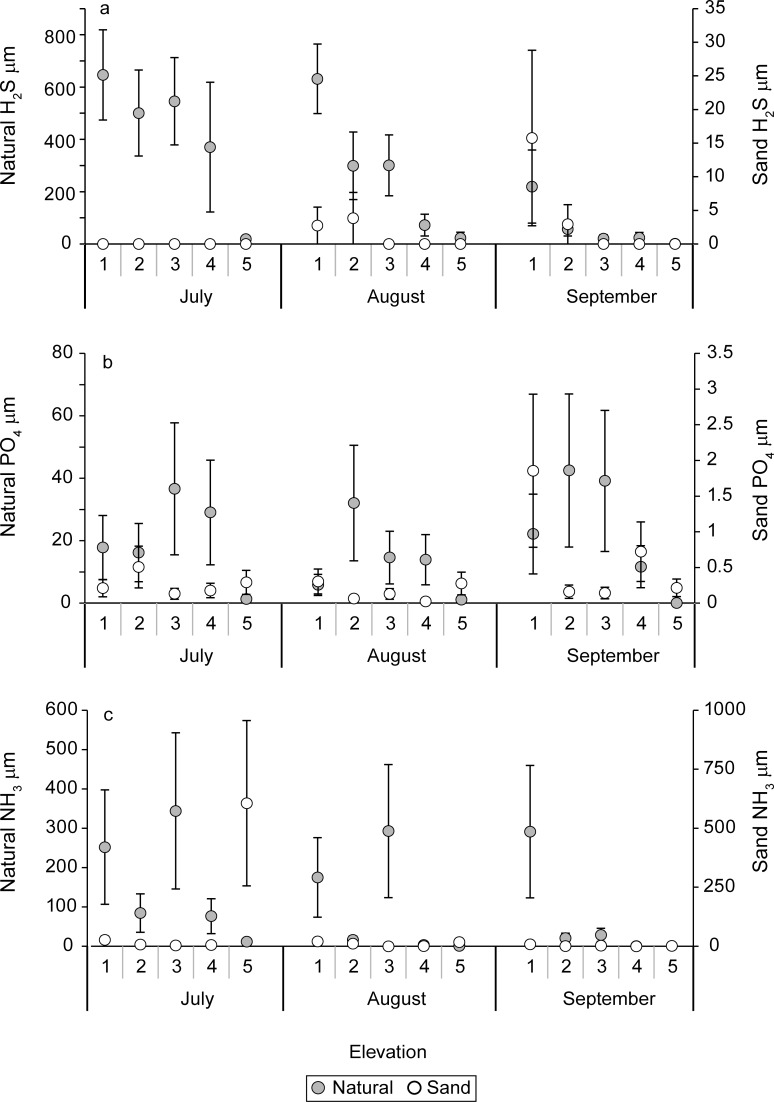
Means and standard errors of pore water (a) hydrogen sulfide (H_2_S, μM), (b) phosphate (PO_4_, μM), and ammonium (NH_4_, μM) concentrations for natural and sand-amended mesocosms at elevation levels 1–5 for July, August, and September.

There were significant interactions between sediment X elevation in sulfide concentrations measured in July and August (P = 0.001 and P = 0.04, respectively). This can be attributed to the lack of any detectable sulfide concentrations in the sand-amended treatments in July and only elevations 3–5 in August and September, compared to nearly all natural sediment treatments having detectable concentrations from July–September. As such, natural sediments had significantly (P < 0.0001) greater sulfides than sand-amended treatments throughout the experiment (Fig 11A). Elevation was a significant (P < 0.005) effect all three months and the sulfides generally decreased from low to high elevations with highest sulfide concentrations at the lowest elevation (i.e., highest inundation regime) in the natural sediment treatments (Fig 11A; Table 4). Low levels of sulfide (< 4 μM) were detected at elevations 1 and 2 in the sand amended treatments and these were lower at elevation 1 than elevation 2 in August.

**Table 4:** ANOVA model results for pore water analytes (H_2_S, PO_4_, NH_4_) with significant (P < 0.05) main effects of elevation. Means ±standard error (SE) are shown for each. Significant sediment X elevation interactions are indicated with an asterisk. Main effects of sediment are indicated. Means with different letters are significantly different (P < 0.05), ns is non-significant.

(μM)	July H_2_S*				July PO_4_*				August H_2_S*				August PO_4_*				Sept H_2_S				Sept PO_4_*				Sept NH_4_			
Soil Matrix	Mean		SE		Mean		SE		Mean		SE		Mean		SE		Mean		SE		Mean		SE		Mean		SE	
Natural	416.4	±	87.0	A	52.7	±	9.6	A	265.3	±	68.9	A	42.6	±	8.0	A	64.2	±	32.4	A	35.0	±	9.9	A	54.8	±	37.3	A
Sand	0.0	±	0.0	B	0.7	±	0.1	B	1.3	±	0.9	B	0.4	±	0.1	B	3.7	±	2.8	B	0.7	±	0.3	B	4.3	±	1.3	B
**Elevation **																												
1	323.3	±	163.9	A	42.6	±	19.2	A	317.2	±	152.7	A	31.0	±	13.7	A	117.6	±	77.6	A	39.0	±	17.5	A	121.8	±	90.3	A
2	250.4	±	133.9	A	38.6	±	17.4	A	151.4	±	87.6	AB	37.0	±	18.4	AB	29.9	±	16.9	AB	29.0	±	16.9	AB	9.8	±	6.3	B
3	272.9	±	143.0	A	33.0	±	17.3	AB	150.3	±	85.0	AB	26.3	±	12.2	AB	10.1	±	8.2	BC	16.8	±	12.5	B	11.5	±	8.2	B
4	185.2	±	138.4	A	17.8	±	10.7	B	36.1	±	24.6	BC	12.3	±	6.5	B	11.9	±	10.4	BC	4.2	±	3.4	B	2.0	±	0.3	B
5	9.3	±	6.9	B	1.4	±	0.5	C	11.4	±	11.0	C	0.7	±	0.3	C	0.3	±	0.3	C	0.2	±	0.1	C	2.7	±	0.5	B

During all three months, there were significant elevation effects (P < 0.001) and sediment X elevation interactions (P < 0.005) for pore water phosphate concentrations. Pore water phosphates were significantly (P < 0.0001) lower in the sand-amended sediments than the natural marsh treatments during July, August, and September (Fig 11B, Table 4). Higher pore water phosphate concentrations were measured at the lower elevations in the natural sediments (Fig 11B). The sand-amended treatments had mostly very low concentrations (< 1 μM) and no consistent patterns with elevation.

There were significant (P ≤ 0.01) main effects of sediment treatment on pore water ammonium all three months with significantly higher concentrations in the natural marsh sediments than the sand-amended ones (Fig 11C, Table 4). In July and August, there was no main effect of elevation and high variability in ammonium concentrations among elevation levels. However, the lowest elevation level (1) had significantly higher ammonium in September, when there was a significant (P = 0.0007) main effect of elevation (Fig 11C). No sediment X elevation interactions were measured for ammonium.

## Discussion

The marsh organ manipulated elevations at a single point in space in an attempt to isolate the effect of hydroperiod from other environmental variables (Fig 1; [28,29,36]). The design allows an approximation of how plants and soils will respond to rising sea levels and increasing periods in inundation [28]. In our study this approach isolated changes in inundation as a driving variable, however, differential effects of other environmental variables such as evapotranspiration, precipitation, and temperature could also contribute to some responses (e.g., productivity) in the experimental pots. In addition, monthly variability in various parameters (e.g. water level, precipitation, temperature) could contribute to plant responses and these might vary from year to year, suggesting longer term experiments might be warranted since our study was only over the course of 6 months. Kirwan and Guntenspergen [36] reported in a 4-year marsh organ study that the biomass of marsh plants receiving different inundation regimes varied from year to year, probably due to environmental variables, but that there was interannual consistency in the optimum flooding for plant productivity and in the pattern of responses among the different elevations.

We provide some background water level data collected at the NOAA Boston Station (https://tidesandcurrents.noaa.gov/waterlevels.html?id=8443970) and meteorological mean rainfall, temperature and winter precipitation from the Plum Island Long Term Ecological Research site (http://pie-lter.ecosystems.mbl.edu/content/data-catalog-research-area) in the S1 File and Table 5 to examine interannual variability associated with the study site. Historic mhw, mean annual rainfall, mean summer and winter temperatures, and mean winter precipitation were reported for the period 1999–2015 and also for the year (2011) of the study. For this period, the mhw was at the high end of the range indicating high water levels in 2011, but the other environmental variables (i.e., annual rainfall, temperature, and winter precipitation) in 2011 were in the midrange for this time period (Table 5). However, due to moderate storms and Hurricane Irene in August 2011, rainfall (about 195 mm) was about two times higher than historic averages (about 100 mm) for this month.

**Table 5:** Mean high water reported at nearby Boston (NOAA station W5-8443970) for 1999–2015, and Plum Island Long Term Ecological Research meteorological data (1999–2015 means for each, annual rainfall, summer temperature (May through September), winter temperature (December–February), and winter precipitation (December–February)).

	1999–2015 mean ± se			1999–2015 Min (year)	1999–2015 Max (year)	2011 Max
Mean High Water (m)	1.382	±	0.013	1.314 (2004)	1.486 (2010)	1.446
Annual Rainfall (m)	1.208	±	0.052	0.856 (2013)	1.456 (2008)	1.255
Summer Temp (°C)	18.4	±	0.01	17.1 (2000)	20.3 (1999)	18.6
Winter Temp (°C)	-1.6	±	0.40	-4.4 (2003)	1.1 (1999)	-2.94
Winter Precip (m)	0.287	±	0.016	0.174 (2012)	0.412 (2003)	0.251

Some interannual variability in absolute plant responses are expected, however interannual consistency in the optimum flooding for productivity and in the pattern of responses have been reported by others [36]. Therefore, the general responses and patterns in this 6 month-long, sand-amendment marsh organ experiment are likely in large part driven by differences in inundation patterns in the marsh organ, and likely reflect marsh responses for a typical growing season in the Northeast USA. *S*. *alterniflora* growth of both the natural and sand-amended treatments was generally greater at intermediate or high elevations. Apparently, the lowest elevations with the highest inundation were sub-optimal for *S*. *alterniflora* growth, as described in earlier studies for *S*. *alterniflora* [28,33] as well as for other plant species (e.g., *Schoenoplectus americanus*, *S*. *patens* [29]; *S*. *maritima* [32]; *S*. *patens*, *Distichlis spicata*, *Juncus gerardii* [45,46,47]). Under high inundation conditions and when there is low sediment supply, plants need to accumulate enough belowground carbon and build peat to keep up with the increased flooding [9,31,48]. If plant productivity is reduced due to the waterlogged conditions, the marsh will erode and this process will contribute to further deterioration. Waterlogging, as simulated by low elevations in our study, can decrease redox potential, increase root anaerobic metabolism, inhibit nitrogen uptake, and stimulate sulfide accumulation [49]. Sulfide concentrations (< 1 mM) were moderate in the natural sediments and low in the sand-amended sediments (< 16 μM) among all 5 elevations in the present study, so other factors (e.g., inhibition of nutrient uptake; root anaerobic metabolism; anoxic conditions) likely contributed to the reduced growth of plants at the lower elevations. At low elevations and high flooding, pore water ammonium concentrations were generally higher, perhaps in part due to low uptake by plants [35]. The high ammonium concentrations under high inundation may be explained by lower uptake by plants or by the expected lower-oxidation status at low elevation levels.

The sand-amended sediments in our study had lower pore water nutrients, higher carbon dioxide efflux, and lower pH. The low nutrients in the sand-amended treatment may have stimulated fine root growth to facilitate nutrient acquisition, and fine roots are a labile carbon source for decomposition. Greater hydraulic conductivity within the sand-amended treatments may cause organic matter to decompose faster than in the natural sediments, which contained more silt and clay particles [50]. Higher soil carbon dioxide emission rates in the sand-amended treatments, especially at the higher elevations (≥ level 3), might also be attributed to more aerobic conditions [51]. The presence of aerobic conditions in the sand-amended treatments at high elevations is further supported by the significantly lower pH, sulfide, phosphate, and ammonium concentrations in the pore water. Reduced sulfur oxidizes in drained marshes causing the acidification of marsh soils; the decreased pH can alter the mobility of phosphorus, iron and other metals [52,53,54]. Ammonium may adsorb onto silts and phosphates which are associated with iron and aluminum oxides under oxidizing conditions as would be the conditions at the higher elevations in the sand-amended treatments in our study [52,53]. Greater evapotranspiration in the high elevation mesocosms may explain the pattern of increasing salinity with increasing elevation in the natural sediment treatments. The reverse pattern in the sand-amended treatments may be explained by the high porosity of the sediments. Seawater exchange at low elevations and rainfall flushing and subsequent leaching at high elevations could explain the pattern of increasing salinity with decreasing elevation in the sand-amended treatments. Rainfall was heaviest in August, the same month the pattern of lowered salinity in the high elevation, sand-amended pots was most evident (Fig 10).

In this study we only tested one plant species, *S*. *alterniflora*, and it is unclear how other marsh plant species might respond to sand amendments under varying inundation regimes. Morphology of marsh roots and rhizomes varies among tidal wetland species (e.g., prevalence of fine and/or coarse roots, average diameters of rhizomes, presence or absence of mycorrhizae), and differences in plant-specific physiology may affect responses to sand amendments and associated changes in biogeochemistry [55,56]. While the coarse root and rhizome abundances of the *S*. *alterniflora* increased in the natural marsh sediments between July and September, the abundances of these structures were similar in the sand-amended treatments over this time period. However, we suspect fine root abundances increased over the course of the experiment in the sand-amended sediments, which resulted in similar total belowground biomass between sediment treatments by the end of the experiment. The natural, higher organic sediments had larger-diameter *S*. *alterniflora* coarse roots in July, perhaps to facilitate oxygen release into the rhizosphere in more reduced sediments compared to the more aerobic, sand-amended sediments [57]. Larger-diameter rhizomes of *S*. *alterniflora* were detected in more impacted and reduced soils than in a reference marsh in the Jamaica Bay Estuary [41]. Plant biomass in natural marshes may actually be less than observed in marsh organs, and Kirwan and Guntenspergen [36] reported approximately 2–4 X greater aboveground biomass (*S*. *americanus*) in their marsh organ mesocosms than natural biomass estimates. In our mesocosm study aboveground biomass of *S*. *alterniflora* ranged from 249–633 g m^-2^ and was similar and overlapped with the range reported for Plum Island plots studied from 1999–2013, where aboveground biomass ranged from 343–1,324 g m^-2^ [28]. Belowground biomass ranged from 3,510–5,591 gm^-2^ in the natural soil mesocosms to 3,030–6,350 g m^-2^ in the sand-amended treatments. The mesocosm belowground estimates are about 30% greater at the high end of the range than estimates for Great Sippewisset marsh (MA), which ranged from 3,212–3,922 g m^-2^ [58]. Marsh mesocosms may alleviate stress from high flow velocities promoting enhanced growth [36]. In addition, growth in the mesocosm may alter lateral root and rhizome extension and artificially promote enhanced belowground growth in the confines of the mesocosm.

Sediment characteristics can be an important consideration in marsh restoration projects. In some locations, non-contaminated sediment from nearby creeks, rather than sand transported from outside the system, might be preferred for a restoration effort. Creek sediment would likely have higher organic matter and nutrients associated with it, which might support more rapid equivalency of plant productivity and microbial processing to reference conditions [59]. Slocum et al. [14] reported an apparent, positive, but short-lived nutrient effect on marshes amended with a nutrient rich sediment slurry originating from the Gulf of Mexico. Vigorous plant growth after the first few years of the study fell and stabilized, but growth was still significantly greater, even after 7 years, than plant growth in areas that did not receive the sediment slurry additions [14]. Unlike sand, natural organic sediment additions might inhibit the oxidation of sulfides, which most likely caused acidic sediment conditions in sand-amended treatments at high elevations in our study. Nevertheless, we observed growth of fine roots, coarse roots, and rhizomes in the sand-amended sediments and no difference in total belowground biomass at the end of 20 weeks. Belowground productivity supports organic matter accumulation and peat buildup in restored marshes, making them more resilient to stressors associated with climate change.

The process of increasing the marsh platform elevation with additions of sand or sediment to natural marsh soils may provide for revegetation, and ultimately for belowground organic matter accumulation, especially if the appropriate thickness and weight of sediment is applied to the landscape (e.g., [14,24,27,60,61]). It may take decades for restored marsh systems to recover to organic carbon and nitrogen accumulation equivalent to natural marsh systems [50,62]; however, increasing marsh elevation and establishing aboveground vegetative cover will help stabilize the marsh system in the short term and afford the system increased resilience to flooding due to sea level rise and storms [14]. Increasing marsh elevation and promoting plant productivity will also provide marsh habitats for salt marsh obligates such as the saltmarsh sparrow (*Ammodramus caudacutus*), whose high marsh habitat is being lost to rising seas [17,63,64]. In conclusion, although significant differences in aboveground biomass and pore water chemistry were measured between the natural and sand-amended sediments, the similar belowground production suggests that sand application to marsh soils will allow for significant root and rhizome growth, which will promote organic matter accumulation and peat build-up. Therefore, application of non-contaminated sediment amendments (e.g., sand, soil slurries, clean dredged sediments) to coastal marsh systems faced with rising seas and increased flooding is an apparently viable climate adaptation option for resource managers.

## Supporting Information

S1 File Supporting data for the results. (XLSX)
